# Coronavirus infection, ER stress, apoptosis and innate immunity

**DOI:** 10.3389/fmicb.2014.00296

**Published:** 2014-06-17

**Authors:** To S. Fung, Ding X. Liu

**Affiliations:** School of Biological Sciences, Nanyang Technological UniversitySingapore, Singapore

**Keywords:** coronavirus, ER stress, apoptosis, signal transduction pathways, proinflammatory cytokines, unfolded protein response

## Abstract

The replication of coronavirus, a family of important animal and human pathogens, is closely associated with the cellular membrane compartments, especially the endoplasmic reticulum (ER). Coronavirus infection of cultured cells was previously shown to cause ER stress and induce the unfolded protein response (UPR), a process that aims to restore the ER homeostasis by global translation shutdown and increasing the ER folding capacity. However, under prolonged ER stress, UPR can also induce apoptotic cell death. Accumulating evidence from recent studies has shown that induction of ER stress and UPR may constitute a major aspect of coronavirus–host interaction. Activation of the three branches of UPR modulates a wide variety of signaling pathways, such as mitogen-activated protein (MAP) kinase activation, autophagy, apoptosis, and innate immune response. ER stress and UPR activation may therefore contribute significantly to the viral replication and pathogenesis during coronavirus infection. In this review, we summarize the current knowledge on coronavirus-induced ER stress and UPR activation, with emphasis on their cross-talking to apoptotic signaling.

## INTRODUCTION

Coronaviruses are a family of enveloped viruses with positive sense, non-segmented, single-stranded RNA genomes. Many coronaviruses are important veterinary pathogens. For example, avian infectious bronchitis virus (IBV) reduces the performance of both meat-type and egg-laying chickens and causes severe economic loss to the poultry industry worldwide (Cavanagh, 2007). Certain coronaviruses, such as HCoV-229E and HCoV-OC43, infect humans and account for a significant percentage of adult common colds (Hamre and Procknow, 1966; Kaye et al., 1972). Moreover, in 2003, a highly pathogenic human coronavirus (SARS-CoV) was identified as the causative agent of severe acute respiratory syndrome (SARS) with high mortality rate and led to global panic (Ksiazek et al., 2003). Later, it was found that the SARS-CoV was originated from bat and likely jumped to humans via some intermediate host (palm civets; Li et al., 2005; Wang and Eaton, 2007). Recently, a live SARS-like coronavirus was isolated from fecal samples of Chinese horseshoe bats, which could use the SARS-CoV cellular receptor – human angiotensin converting enzyme II (ACE2) for cell entry (Ge et al., 2013). This indicates that an intermediate host may not be necessary and direct human infection by some bat coronaviruses is possible. Moreover, a novel human coronavirus – the Middle East respiratory syndrome coronavirus (MERS-CoV), emerged in Saudi Arabia in September 2012 (de Groot et al., 2013). Although the risk of sustained human-to-human transmission is considered low, infection of MERS-CoV causes ~50% mortality in patients with comorbidities (Graham et al., 2013). Initial studies had pointed to bats as the source of MERS-CoV (Annan et al., 2013); however, accumulating evidence strongly suggested the dromedary camels to be the natural reservoirs and animal source of MERS-CoV (Hemida et al., 2013; Alagaili et al., 2014). Thus, coronaviruses can cross the species barrier to become lethal human pathogens, and studies on coronaviruses are both economically and medically important.

Taxonomically, the family *Coronaviridae* is classified into two subfamilies, the *coronavirinae* and the *torovirinae*. The *coronavirinae* is further classified into three genera, namely the Alphacoronavirus, Betacoronavirus, and Gammacoronavirus (Masters, 2006). The classification was originally based on antigenic relationships and later confirmed by sequence comparisons of entire viral genomes (Gorbalenya et al., 2004). Almost all Alphacoronaviruses and Betacoronaviruses have mammalian hosts, including humans. In contrast, Gammacoronaviruses have mainly been isolated from avian hosts.

Morphologically, coronaviruses are spherical or pleomorphic in shape with a mean diameter of 80–120 nm. They are characterized by the large (20 nm) “club-like” projections on the surface, which are the heavily glycosylated trimeric spike (S) proteins (Masters, 2006). Two additional structural proteins are found on the envelope. The abundant membrane (M) proteins give the virion its shape, whereas the small envelope (E) proteins play an essential role during assembly (Sturman et al., 1980; Liu and Inglis, 1991). Inside the envelope, the helical nucleocapsid is formed by binding of the nucleocapsid (N) proteins on the genomic RNA in a beads-on-a-string fashion. The genome, ranging from 27,000 to 32,000 nucleotides in size, is the largest RNA genomes known to date.

**FIGURE 1:**
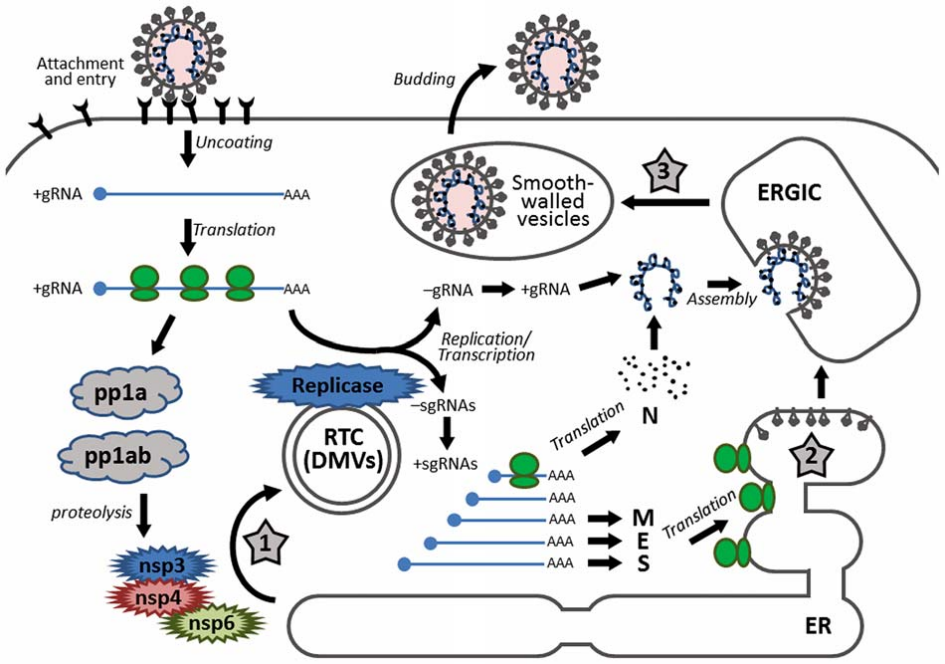
**Schematic diagram showing the replication cycle of coronavirus and the stages in which ER stress may be induced during coronavirus infection.** Infection starts with receptor binding and entry by membrane fusion. After uncoating, the genomic RNA is used as a template to synthesize progeny genomes and a nested set of subgenomic RNAs. The replication transcription centers are closely associated with DMVs, which are proposed to be adopted from the modified ER, possibly by the combined activities of non-structural proteins nsp3, nsp4, and nsp6. The S, E, and M proteins are synthesized and anchored on the ER, whereas the N protein is translated in the cytosol. Assembly takes place in the ERGIC and mature virions are released via smooth-walled vesicles by exocytosis. The three stages that presumably induce ER stress are highlighted with numbered star signs, namely: (1) formation of DMVs, (2) massive production and modification of structural proteins, and (3) depletion of ER membrane during budding.

Coronavirus infection starts with receptor binding via the S protein (**Figure 1**). The S proteins of most coronaviruses are cleaved by host protease into two functional subunits: an N-terminal receptor binding domain (S1) and a C-terminal domain (S2) responsible for membrane fusion (Huang et al., 2006; Qiu et al., 2006; Yamada et al., 2009). The interaction between the cell surface receptor and the S1 subunit is the major determinant of the tropism of coronaviruses (Kuo et al., 2000). Upon receptor binding of S1, a conformational change is triggered in the S2 subunit, exposing its hidden fusion peptide for insertion into the cellular membrane. This is followed by the packing of the two heptad repeats in the three monomers into a six-helix bundle fusion core. This close juxtaposition of the viral and cellular membrane enables fusion of the lipid bilayers, and the viral nucleocapsid is thus delivered into the cytoplasm (Masters, 2006).

After uncoating, the genomic RNA first acts as an mRNA for translation of the replicase polyprotein. The replicase gene consists of two open reading frames (ORF1a and ORF1b). Translation of ORF1a produces the polyprotein 1a (pp1a). Meanwhile, a ribosomal frameshifting occurs at the junction of ORF1a and ORF1b, allowing translation to continue onto ORF1b, producing a larger polyprotein 1ab (pp1ab; Brierley et al., 1987). Autoproteolytic cleavage of pp1a produces 11 non-structural proteins (nsp1–nsp11), while cleavage of pp1ab produces 15 non-structural proteins (nsp1–nsp10 and nsp12–nsp16). The functions of these nsps are partially understood. Particularly, the autoproteolytic cleavage relies on nsp3 (a papain-like proteinase) and nsp5 (the main proteinase), whereas the RNA-dependent RNA polymerase (RdRp) is contained within nsp12 (Baker et al., 1993; Lu et al., 1995a).

Using the genomic RNA as a template, the replicase then synthesizes the negative sense genomic RNAs, which are used as templates for synthesizing progeny positive sense RNA genomes. On the other hand, through discontinuous transcription of the genome, the replicase synthesizes a nested set of subgenomic RNAs (sgRNAs; Sawicki et al., 2007). Replication and transcription of the coronavirus genome involve the formation of the replication/transcription complexes (RTCs), which are anchored to the intracellular membranes via the multi-spanning transmembrane proteins nsp3, nsp4, and nsp6 (Oostra et al., 2007). Also, inside the infected cells, coronaviruses induce modification of the intracellular membrane network and formation of the double membrane vesicles (DMVs; Knoops et al., 2008). Several studies have shown that the DMVs are closely associated with the coronavirus RTCs and the *de novo* synthesized viral RNAs (Gosert et al., 2002; Snijder et al., 2006).

The sgRNAs are translated into structural proteins and accessory proteins. Transmembrane structural proteins (S, M, and E) are synthesized, inserted, and folded in the endoplasmic reticulum (ER) and transported to the ER–Golgi intermediate compartment (ERGIC). The N proteins are translated in the cytoplasm and encapsidate the nascent progeny genomic RNA to form the nucleocapsids. Virion assembly occurs in the ERGIC and is likely to be orchestrated by the M protein through protein–protein interactions (Masters, 2006).

The virions budded into the ERGIC are exported through secretory pathway in smooth-wall vesicles, which ultimately fuse with the plasma membrane and release the mature virus particles (Krijnse-Locker et al., 1994). For some coronaviruses, a portion of the S protein escapes from viral assembly and is secreted to the plasma membrane. These S proteins cause fusion of the infected cell with neighboring uninfected cells, resulting in the formation of a large, multinucleated cell known as a syncytium, which enables the virus to spread without being released into the extracellular space (Masters, 2006).

**FIGURE 2:**
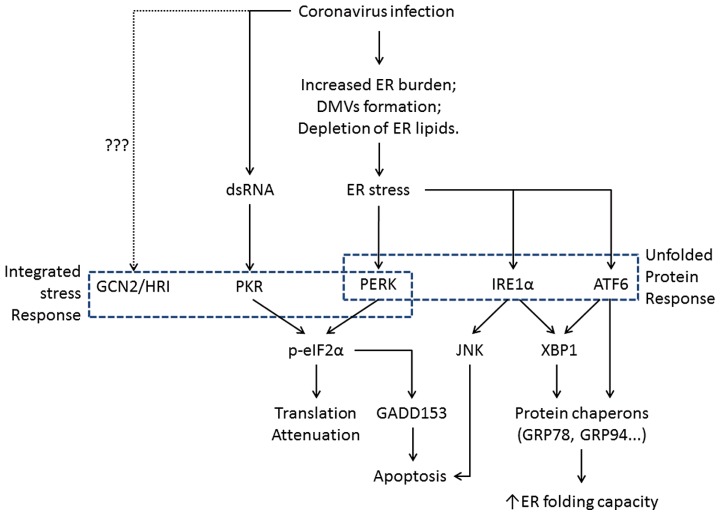
**Flowchart showing the induction of ER stress and its physiological outcomes during coronavirus infection.** The integrated stress response pathways (including PERK) trigger translation shutdown and modulate apoptosis. The ATF6 pathway enhances the ER folding capacity, and the IRE1 pathway affects both ER folding and apoptosis induction. Pointed arrows indicate activation. The dotted line suggests uncharacterized function of GCN2 and HRI during coronavirus infection.

In eukaryotic cells, ER is the major site for synthesis and folding of secreted and transmembrane proteins. The amount of protein entering the ER can vary substantially under different physiological states and environmental conditions. When protein synthesis surpasses the folding capacity, unfolded proteins accumulate in the ER and lead to ER stress. ER stress can also be activated by excessive lipids or pro-inflammatory cytokines (Kharroubi et al., 2004; Pineau et al., 2009). To maintain homeostasis, cells have evolved signaling pathways that are collectively known as the unfolded protein response (UPR; Ron and Walter, 2007). The UPR signaling starts with the unfolded proteins activating the three ER stress transducers: PKR-like ER protein kinase (PERK), activating transcriptional factor-6 (ATF6), or inositol-requiring protein-1 (IRE1; **Figure 2**). Once activated, these sensors transmit the signal across the ER membrane to the cytosol and the nucleus, and the cell responds by lowering the protein synthesis and increasing the ER folding capacity. If homeostasis cannot be re-established, apoptosis is induced for the benefit of the entire organism (Tabas and Ron, 2011).

In this review, current studies on the involvement of the UPR in coronavirus infection and pathogenesis will be summarized. The role of UPR activation in host response, in particular the induction of apoptosis, will also be reviewed.

## CORONAVIRUS INFECTION AND ER STRESS

Global proteomic and microarray analyses have shown that the expression of several genes related to the ER stress, such as glucose-regulated protein 94 (GRP94) and glucose-regulated protein 78 (GRP78, also known as immunoglobulin heavy chain-binding protein, or BiP), is up-regulated in cells infected with SARS-CoV or in cells overexpressing the SARS-CoV S2 subunit (Jiang et al., 2005; Yeung et al., 2008). Using a luciferase reporter system, Chan et al. (2006) found that both GRP94 and GRP78 were induced in SARS-CoV-infected FRhK4 cells. Consistently, the mRNA level of homocysteine-inducible, ER stress-inducible, ubiquitin-like domain member 1 (HERPUD1), an ER stress marker, was up-regulated in L cells infected with mouse hepatitis virus (MHV) or SARS-CoV (Versteeg et al., 2007). Data from this group have shown a similar induction of ER stress in IBV-infected Vero, H1299, and Huh-7 cells (unpublished observations). Although no parallel studies have been performed on Alphacoronaviruses, it is likely that all three genera of coronaviruses may induce ER stress in the infected cells. Current evidence suggests the following three main mechanisms.

### FORMATION OF DOUBLE MEMBRANE VESICLES

It is well-known that the replication of many plus-stranded RNA viruses induces modification of cellular membranes (Miller and Krijnse-Locker, 2008). Among them, coronaviruses have been shown to induce the formation of DMVs in infected cells (David-Ferreira and Manaker, 1965). Based on immunocytochemistry electron microscopy data, the DMVs co-localize with coronavirus major replicase proteins and are presumably the sites where coronavirus RTCs are located (Gosert et al., 2002; Snijder et al., 2006). Indeed, DMVs are induced in HEK293T cells co-expressing the SARS-CoV nsp3, nsp4, and nsp6, which are all multispanning transmembrane non-structural proteins (Angelini et al., 2013). There have been different perspectives regarding the origin of the coronavirus-induced DMVs. The late endosomes, autophagosomes, and the early secretary pathway have all been implicated as the membrane source of DMVs (van der Meer et al., 1999; Prentice et al., 2004; Verheije et al., 2008). Also, co-localization has been observed between SARS-CoV non-structural proteins and protein disulfide isomerase (PDI), an ER marker (Snijder et al., 2006). Using high-resolution electron tomography, Knoops et al. (2008) have shown that infection of SARS-CoV reorganizes the ER into a reticulovesicular network, which consists of convoluted membranes and interconnected DMVs. Recently, Reggiori et al. (2010) have proposed a model in which coronaviruses hijack the EDEMosomes to derive ER membrane for DMV formation. The EDEMosomes are COPII-independent vesicles that export from the ER, which are normally used to fine-tune the level of ER degradation enhancer, mannosidase alpha-like 1 (EDEM1), a regulator of ER-associated degradation (ERAD; Calì et al., 2008). It has been demonstrated that MHV infection causes accumulation of EDEM1 and osteosarcoma amplified 9 (OS-9, another EDEMosome cargo), and that both EDEM1 and OS-9 co-localize with the RTCs of MHV (Reggiori et al., 2010). These results thus add mechanical evidence to support the ER-origin of the coronavirus-induced DMVs.

### GLYCOSYLATION OF CORONAVIRAL STRUCTURAL PROTEINS

Except for the N protein, all coronavirus structural proteins are transmembrane proteins synthesized in the ER. The M protein, which is the most abundant component of the virus particle, is known to undergo either O-linked (for most betacoronaviruses) or N-linked (for all alpha- and gammacoronaviruses) glycosylation in the ER (Jacobs et al., 1986; Cavanagh and Davis, 1988; Nal et al., 2005). The glycosylation of M protein is proposed to play a certain function in alpha interferon (IFN) induction and *in vivo* tissue tropism (Charley and Laude, 1988; Laude et al., 1992; de Haan et al., 2003). The pre-glycosylated S monomers are around 128–160 kDa, whereas sizes can reach 150–200 kDa post-glycosylation (exclusively N-linked), indicating that the S protein is highly glycosylated (Masters, 2006). At least for transmissible gastroenteritis coronavirus (TGEV), glycosylation is presumed to facilitate monomer folding and trimerization (Delmas and Laude, 1990). Moreover, the glycans on SARS-CoV S proteins have been shown to bind C-type lectins DC-SIGN (dendritic cell-specific intercellular adhesion molecule-3-grabbing non-integrin) and L-SIGN (liver lymph node-specific intercellular adhesion molecule-3-grabbing non-integrin), which can serve as alternative receptors for SARS-CoV independent of the major receptor ACE2 (Han et al., 2007). The folding, maturation, and assembly of the gigantic S trimeric glycoprotein rely heavily on the protein chaperons inside the ER, such as calnexin. In fact, the N-terminal part of the S2 domain of SARS-CoV S protein has been found to interact with calnexin, and knock-down of calnexin decreases the infectivity of pseudotyped lentivirus carrying the SARS-CoV S protein (Fukushi et al., 2012). Also, treatment of α-glucosidase inhibitors, which inhibit the interactions of calnexin with its substrates, dose dependently inhibits the incorporation of S into pseudovirus and suppresses SARS-CoV replication in cell cultures (Fukushi et al., 2012). During coronavirus replication, massive amount of structural proteins is synthesized to assembly progeny virions. The production, folding, and modification of these proteins undoubtedly increase the workload of the ER.

### DEPLETION OF ER LIPID DURING THE BUDDING OF VIRIONS

Budding of coronaviruses occurs in the ERGIC, which is a structural and functional continuance of the ER. Thus, the release of mature virions by exocytosis in effect depletes the lipid component of the ER. Taken together, coronavirus infection results in: (1) massive morphological rearrangement of the ER; (2) significant increase ER burden for protein synthesis, folding and modification; and (3) extensive depletion of ER lipid component. These factors together may contribute to the coronavirus-induced ER stress.

In the following sections, the activation of the three individual branches of the UPR by coronavirus infection will be discussed in detail.

## THE PERK BRANCH OF UPR

### PERK-EIF2α-ATF4 SIGNALING PATHWAY

The PERK branch of the UPR is believed to be activated first in response to ER stress (Szegezdi et al., 2006). Activation of PERK begins with the dissociation from ER chaperon BiP, followed by oligomerization and auto-phosphorylation. Activated PERK then phosphorylates the α-subunit of eukaryotic initiation factor 2 (eIF2α). Phosphorylated eIF2α forms a stable complex with and inhibits the turnover of eIF2B, a guanine nucleotide exchange factor that recycles inactive eIF2-GDP to active eIF2-GTP. This results in a general shutdown of cellular protein synthesis and reduces the protein flux into the ER (Ron and Walter, 2007). Besides PERK, three other kinases are known to phosphorylate eIF2α, namely the protein kinase RNA-activated (PKR), heme-regulated inhibitor kinase (HRI), and general control non-derepressible-2 (GCN2; Ron and Walter, 2007). PKR is induced by IFN and activated by the binding of double-stranded RNA (dsRNA) after virus infection (Clemens and Elia, 1997). HRI is activated in red blood cells and hepatocytes by low levels of heme (McEwen et al., 2005). GCN2 senses amino acid deficiency and is activated via binding to uncharged transfer RNAs (Sood et al., 2000). Due to common outcome (eIF2α phosphorylation and translation suppression), activation of these kinases is collectively known as the integrated stress response (ISR; Ron and Walter, 2007).

**FIGURE 3:**
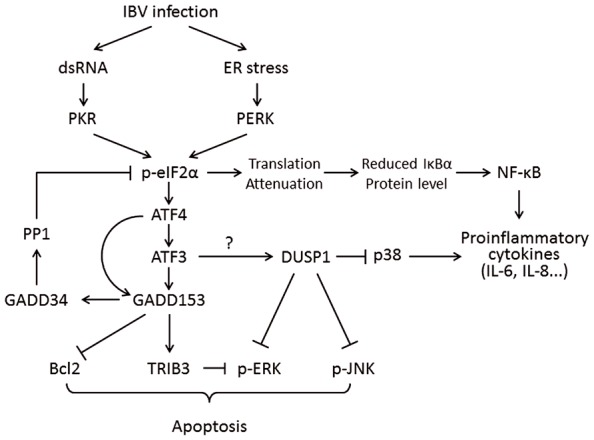
**Working model of PKR/PERK-eIF2α-ATF4-GADD153 pathway activation during coronavirus infection, using IBV as an example.** Phosphorylation of eIF2α by PERK and PKR induces the expression of ATF4, ATF3, and GADD153. GADD153 exerts its pro-apoptotic activities via suppressing Bcl2 and ERKs by inducing TRIB3. The potential induction of DUSP1 by ATF3 may modulate phosphorylation of p38 and JNK, thus regulating IBV-induced apoptosis and cytokine production. The translation attenuation due to eIF2α activation can also lead to reduced inhibition of IκBα on NF-κB, which in turn promote cytokine production. Pointed arrows indicate activation, and blunt-ended lines indicate inhibition. The question mark indicates hypothetical mechanism.

Interestingly, the mRNAs of certain genes contain small ORFs in their 5′ UTR and bypass the eIF2α-dependent translation block. One of these is the activating transcription factor 4 (ATF4), which is preferentially translated under ISR. ATF4 in turn transactivates genes involved in amino acid metabolism, redox reactions, and stress response. One of ATF4’s target genes is the growth arrest and DNA damage-inducible protein 153 (GADD153, also known as C/EBP homologous protein, or CHOP). GADD153 induces the growth arrest and DNA damage-inducible protein 34 (GADD34), which recruits protein phosphatase 1 (PP1) to dephosphorylate eIF2α and release the translation block. To this end, if ER stress is resolved, normal protein synthesis can be resumed. However, if ER stress persists, GADD153 can induce apoptosis by suppressing the anti-apoptotic protein B-cell lymphoma 2 (Bcl-2) and inducing the pro-apoptotic proteins such as Bcl-2-interacting mediator of cell death (Bim; Puthalakath et al., 2007). GADD153 also activates ER oxidoreductin-1α (ERO1α), which encodes an ER oxidase. The increase protein influx to a hyper-oxidizing ER aggravates ER stress and induces apoptosis (Marciniak et al., 2004; **Figure 3**).

### INVOLVEMENT OF THE PERK PATHWAY DURING VIRAL INFECTIONS

Translation attenuation has been widely observed as a defensive mechanism of the host cells against viral infection. By reducing the translation of viral proteins, virus replication is hampered and the spread of infection is limited, giving enough time for the immune system to initiate effective antiviral responses. Among the four eIF2α kinases, PKR, due to its IFN-inducible nature and specific recognition of viral dsRNAs, plays an especially important role in inducing translation attenuation in virus-infected cells (He, 2006). It is therefore not surprising that viruses have evolved various mechanisms to counteract PKR. For example, the non-structural 5A (NS5A) protein of hepatitis C virus directly interacts with the catalytic site of PKR, whereas the NS1 protein in the influenza A virus binds to dsRNAs and thus blocks PKR activation (Lu et al., 1995b; Gale et al., 1997).

During virus infection, massive production of viral proteins can overload the folding capacities of ER and lead to activation of another eIF2α kinase – PERK. Activation of PERK has been observed in cells infected with various DNA and RNA viruses, such as vesicular stomatitis virus, bovine viral diarrhea virus and herpes simplex virus 1 (HSV1), to name just a few (Jordan et al., 2002; Baltzis et al., 2004; Cheng et al., 2005). However, similar to PKR, viruses have adopted counter measures to inhibit PERK-mediated translation attenuation. For example, the E2 protein of hepatitis C virus (HCV) and the glycoprotein gB of HSV1 bind to PERK and inhibit its kinase activity to rescue translation (Pavio et al., 2003; Mulvey et al., 2007).

### ACTIVATION OF PERK PATHWAY DURING CORONAVIRUSES INFECTION AND ITS INVOLVEMENT IN CORONAVIRUS-INDUCED APOPTOSIS

There have been diverging results on the activation of PKR and/or PERK during coronavirus infection. In an early study, it has been found that there is minimal transcriptional activation of PKR and another IFN-stimulated gene, 2′5′-oligoadenylate synthetase (OAS) in cells infected with MHV-1 (Zorzitto et al., 2006). In a separate study, phosphorylation of PKR and eIF2α was also not observed in MHV A59-infected cells (Ye et al., 2007). However, Bechill et al. (2008) have detected significant eIF2α phosphorylation and up-regulation of ATF4 in cells infected with MHV A59, although no induction of GADD153 and GADD34 was observed. It has been suggested that due to the lack of GADD34-mediated eIF2α dephosphorylation, MHV infection induces sustained translation repression of most cellular proteins (Bechill et al., 2008). However, the translation of MHV mRNAs seems to be resistant to eIF2α phosphorylation, and the detailed mechanisms for such evasion are yet to be investigated. As for SARS-CoV, PKR, PERK, and eIF2α phosphorylation are readily detectable in virus-infected cells (Krähling et al., 2009). However, knock-down of PKR using specific morpholino oligomers did not affect SARS-CoV-induced eIF2α phosphorylation but significantly inhibited SARS-CoV-induced apoptosis (Krähling et al., 2009). It is possible that eIF2α is phosphorylated by PERK in SARS-CoV-infected cells, but similar loss-of-function experiments have not been performed, although overexpression of SARS-CoV accessory protein 3a has been shown to activate the PERK pathway (Minakshi et al., 2009).

The discrepancy regarding the activation of PKR/PERK during coronavirus infection may be a result from the different cell culture systems and virus strains used. The interpretation is further complicated by the IFN-inducible nature of PKR. It is generally believed that coronaviruses are poor type I IFN inducers *in vitro* (Garlinghouse et al., 1984; Spiegel et al., 2005; Roth-Cross et al., 2007), although the IFN response may be essential for antiviral activities *in vivo* (Ireland et al., 2008). Moreover, it is known that coronaviruses employ multiple mechanisms to antagonize the IFN response. For example, the nsp16 has been shown to utilize the 2′-*O*-methyltransferase activity to modify coronavirus mRNAs, so as to evade from the cytosolic RNA sensor melanoma differentiation-associated protein 5 (MDA5) and type I IFN induction (Roth-Cross et al., 2008; Züst et al., 2011). Furthermore, the activities of several IFN-induced genes (ISGs) have also been shown to be modulated by coronaviruses during infection. For instance, Zhao et al. (2012) have demonstrated that the MHV accessory protein ns2 cleaves 2′,5′-oligoadenylate, the product of an ISG called OAS. This results in the suppression of the cellular endoribonuclease RNase L activity and facilitates virus replication *in vitro* and *in vivo* (Zhao et al., 2011, 2012). Thus, similar uncharacterized mechanisms may be used by MHV and other coronaviruses to block the activation and/or downstream signaling of PKR. In this regard, the activation of PERK via ER stress seems to be an alternative pathway to activate eIF2α, although coronaviruses may counteract by directly targeting eIF2α, as described below.

Studies done by this group have shown that, phosphorylation of PKR, PERK, and eIF2α was detectable at early stage of IBV infection (0–8 hpi) but diminished quickly later (Wang et al., 2009; Liao et al., 2013). The rapid de-phosphorylation of eIF2α is likely due to the accumulation of GADD34, which is a component of the PP1 complex and a downstream target gene induced by GADD153 (Wang et al., 2009). Despite of the rapid de-phosphorylation of eIF2α, significant induction of GADD153 was observed at late stage of infection (16–24 h) at both mRNA and protein levels (Liao et al., 2013). The up-regulation of GADD153 was likely mediated by both PKR and PERK, since knock-down of either PKR or PERK by siRNA reduces IBV-induced GADD153 (Liao et al., 2013). The up-regulation of GADD153 promotes apoptosis in IBV-infected cells, possibly via inducing the pro-apoptotic protein tribbles-related protein 3 (TRIB3) and suppressing the pro-survival kinase extracellular signal-related kinase (ERK; Liao et al., 2013). Based on the findings so far obtained, it is safe to conclude that the PERK/PKR-eIF2α-ATF4-GADD153 pathway is activated by some, but not all, coronaviruses. In the infected cells, this pathway is activated at an early stage but quickly modulated by feedback de-phosphorylation. The PERK/PKR-eIF2α-ATF4-GADD153 most likely plays a pro-apoptotic function during coronavirus infection.

### INTEGRATED STRESS RESPONSE PATHWAYS AND INNATE IMMUNITY

Several recent studies have demonstrated the critical roles of cellular stress response pathways in modulating the innate immune activation (Cláudio et al., 2013). One of the key regulators that bridge stress and innate immunity is GADD34, a negative regulator of eIF2α activation. It has been shown that when stimulated with polyriboinosinic:polyribocytidylic acid (polyI:C), the integrated stress response pathways were activated in dendritic cells (DCs), leading to up-regulation of ATF4 and GADD34 (Clavarino et al., 2012). Interestingly, GADD34 expression did not significantly affect protein synthesis in DCs, but was shown to be crucial for the production of interferon β (IFN-β) and pro-inflammatory cytokines interleukin-6 (IL-6; Clavarino et al., 2012). In contrast, GADD34 has also been shown to specify PP1 to dephosphorylate the TGF-β-activated kinase 1 (TAK1), thus negatively regulating the toll-like receptor (TLR) signaling and pro-inflammatory cytokines [IL-6 and TNF-α (Tumor necrosis factor alpha)] production in macrophages (Gu et al., 2014). The functional disparities of GADD34 in DCs and macrophages indicate that the integrated stress response may be regulated by some other signaling pathways, resulting in cell-type specific outcomes in the innate immune activation. Since GADD34 induction was readily observed in cells infected with IBV (Wang et al., 2009), it will be intriguing to ask whether GADD34 also contributes to IBV-induced pro-inflammatory cytokine production, and to determine potential cross-talks between the PERK pathway and innate immune activation during IBV infection.

The massive production of pro-inflammatory cytokines (cytokine storm) has been associated with the immunopathogenesis and high mortality rate of SARS-CoV (Perlman and Dandekar, 2005). The transcription factor nuclear factor kappa-light-chain-enhancer of activated B cells (NF-κB) is a master regulator of pro-inflammatory response and innate immunity (Hayden and Ghosh, 2012). It has been well established that NF-κB is required for the induction of pro-inflammatory cytokines (such as IL-6 and IL-8) and the early expression of IFN-β during RNA virus infection (Libermann and Baltimore, 1990; Kunsch and Rosen, 1993; Wang et al., 2010; Balachandran and Beg, 2011; Basagoudanavar et al., 2011). Interestingly, induction of TNF-α, IL-6, and IL-8 has been detected in cells overexpressing the spike protein of SARS-CoV via the NF-κB pathway (Wang et al., 2007; Dosch et al., 2009). Thus, it is intriguing to consider the involvement of ER stress in activating the NF-κB pathway during coronavirus infection. In its inactive form, NF-κB is sequestered in the cytoplasm by inhibitor of NF-κB alpha (IκBα), which masks the nuclear localization signal of NF-κB (Karin and Ben-Neriah, 2000). The basal level of IκBα is maintained by constitutive synthesis and degradation of the protein (Kanarek et al., 2010). Under various stress conditions, phosphorylation of eIF2α leads to global translation repression and a net decrease in IκBα protein level (Jiang et al., 2003). This then results in the activation of NF-κB and induction of pro-inflammatory response (**Figure 3**). Nonetheless, further studies are needed to characterize the actual contributions of ER stress in NF-κB-mediated cytokine induction during coronavirus infection.

Previous study done by this group has shown that infection of IBV induced the production of IL-6 and IL-8, which was dependent on the phosphorylation of MAP kinase p38 (Liao et al., 2011). Interestingly, a protein phosphatase called dual-specificity phosphatase 1 (DUSP1) was also up-regulated in IBV-infected cells and dephosphorylated p38 to modulate pro-inflammatory cytokine production (Liao et al., 2011). Previous studies have shown that the mRNA and protein levels of DUSP1 are modulated by ER stress (Boutros et al., 2008; Li et al., 2011). ER stress-induced DUSP1 up-regulation is likely to be mediated by ATF3 in the PERK pathway, since knock-down of ATF3 significantly reduced DUSP1 induction in cells under ER stress (Gora et al., 2010). Thus, it is possible that IBV infection activates the PERK branch of UPR to induce DUSP1 expression, which in turn dephosphorylates p38 to modulate IBV-induced pro-inflammatory cytokine production (**Figure 3**).

Besides p38, DUSP1 has also been shown to dephosphorylate c-Jun N-terminal kinase (JNK) and ERK (Sun et al., 1993; Franklin and Kraft, 1997). It has been long proposed that ERK phosphorylation promotes cell survival, whereas prolonged JNK and p38 phosphorylation is linked to the induction of apoptosis (Xia et al., 1995). Thus, the induction of DUSP1 by ER stress in coronavirus-infected cells may also contribute to virus-induced apoptosis via modulation of the MAP kinase pathways.

## THE IRE1 BRANCH OF UPR

### IRE1-XBP1 SIGNALING PATHWAY

**FIGURE 4:**
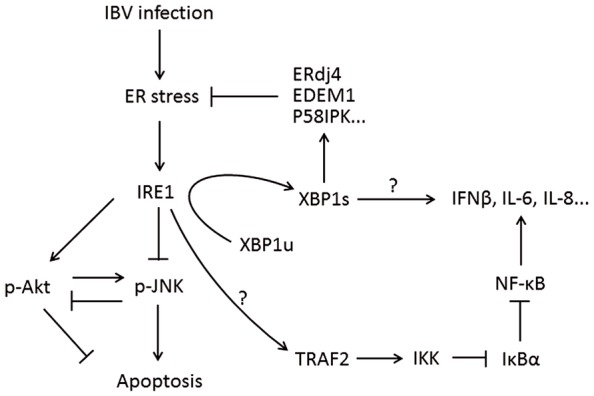
**Working model of IRE1-XBP1 signaling pathway during coronavirus infection, using IBV as an example.** IRE1 mediates XBP1 splicing, which up-regulates UPR target genes to restore ER stress, and the spliced XBP1 may also modulate the IFN and cytokine secretion. IRE1 activation modulates the phosphorylation of Akt and JNK, thus affecting IBV-induced apoptosis. IRE1 is also responsible for basal activity of IKK, which phosphorylates IκBα to remove its inhibition on NF-κB, thus facilitating the production of type I IFN and pro-inflammatory cytokines. Pointed arrows indicate activation, and blunt-ended lines indicate inhibition. The question mark indicates hypothetical mechanism.

The IRE1-XBP1 branch of the UPR is evolutionarily conserved from yeast to humans. In response to unfolded proteins, IRE1 undergoes oligomerization (Bertolotti et al., 2000). This results in trans-autophosphorylation of the kinase domain and the activation of IRE1’s RNase domain. So far, the only known substrate for IRE1 RNase activity is the mRNA of the X box binding protein 1 (XBP1) gene (Yoshida et al., 2001a; Calfon et al., 2002). IRE1 cuts the XBP1 mRNA twice, removing a 26-nucleotide intron to form a frameshifted transcript, the spliced XBP1 (XBP1s). Whereas the unspliced XBP1 mRNA (XBP1u) encodes an inhibitor of the UPR, XBP1s encode a potent transcriptional activator, which translocates to the nucleus and enhances the expression of many UPR genes, including those encoding molecular chaperones and proteins contributing to ER-associated degradation (Ng et al., 2000; Lee et al., 2003; **Figure 4**).

Apart from the XBP1 pathway, activated IRE1 has been shown to recruit TNF receptor-associated factor 2 (TRAF2) and induce apoptosis by activating the JNK (Urano et al., 2000). This IRE1-JNK pathway is independent of IRE1’s RNase activity, but it requires IRE1’s kinase domain and involves TRAF2-dependent activation of caspase-12 (Yoneda et al., 2001). Moreover, one recent study has demonstrated that the IRE1-JNK pathway is required for autophagy activation after pharmacological induction of ER stress. It was found that the kinase domain but not the RNase activity of IRE1 was required, and treatment of a JNK inhibitor (SP600125) abolished autophagosome formation after ER stress (Ogata et al., 2006). Therefore, the IRE1 branch of UPR is closely associated with the JNK pathway and involved in JNK-mediated apoptosis and autophagy signaling.

### ACTIVATION OF THE IRE1 PATHWAY DURING CORONAVIRUSES INFECTION

The involvement of IRE1-XBP1 pathway during coronavirus infection has been investigated by several studies, using MHV as a model. Either MHV infection or overexpression of the MHV S protein (but not other structural proteins) induces XBP1 mRNA splicing (Versteeg et al., 2007; Bechill et al., 2008). However, although XBP1 mRNA is efficiently spliced, the protein product of spliced XBP1 cannot be detected in either the whole cell lysate or the nuclear fraction. Moreover, UPR downstream genes known to be activated by XBP1s, such as ER DNA J domain-containing protein 4 (ERdj4), EDEM1, and protein kinase inhibitor of 58 kDa (p58^IPK^), are not significantly induced after infection (Bechill et al., 2008). Using a luciferase reporter system, it is shown that MHV infection does not inhibit transactivation of unfolded protein response element (UPRE) and ER stress response element (ERSE) promoter by XBP1s. Because MHV infection is associated with persistent eIF2α phosphorylation and host translational repression, it is likely that failure to translate the XBP1s protein may be the main reason why activation of the IRE1 branch does not occur even though XBP1 mRNA splicing is observed. On the other hand, although SARS-CoV belongs to the same genera of Betacoronavirus as MHV, neither infection with SARS-CoV nor overexpression of SARS-CoV S protein induces XBP1 mRNA splicing (Versteeg et al., 2007; DeDiego et al., 2011). It is possible that other viral proteins of SARS-CoV (such as the E protein mentioned below), function as an antagonist of IRE1-XBP1 activation.

Result from this group has also shown that the IRE1-XBP1 pathway is activated in cells infected with IBV. In IBV-infected Vero cells, significant splicing of XBP1 mRNA was detected starting from 12 to 16 h post-infection till the late stage of infection. The mRNA levels of XBP1 effector genes (EDEM1, ERdj4, and p58^IPK^) were up-regulated in IBV-infected Vero cells. The activation of IRE1-XBP1 pathway was also detectable, though at a lower level, in other cell lines such as H1299 and Huh-7 cells. Treatment of IRE1 inhibitor effectively blocked IBV-induced XBP1 mRNA splicing and effector genes up-regulation in a dosage-dependent manner. Consistently, knockdown of IRE1 inhibited IBV-induced XBP1 mRNA splicing, whereas overexpression of wild-type IRE1 (but not its kinase dead or RNase domain deleted mutants) enhanced IBV-induced XBP1 mRNA splicing. These results suggest that the IRE1-XBP1 pathway is indeed activated in cells infected with IBV. Interestingly, an earlier onset and more significant apoptosis induction in IRE1-knockdown IBV-infected cells was observed, which is associated with hyper-phosphorylation of pro-apoptotic kinase JNK and hypo-phosphorylation of pro-survival kinase RAC-alpha serine/threonine-protein kinase (Akt). Taken together, IRE1 may modulate IBV-induced apoptosis and serve as a survival factor during coronavirus infection.

Interestingly, a recent report by DeDiego et al. (2011) demonstrates that the coronavirus E protein may modulate the IRE1-XBP1 pathway. Using a recombinant SARS-CoV that lacks the E protein (rSARS-CoV-ΔE), it is found that both XBP1 splicing and induction of UPR genes significantly increase in the absence of E protein. Moreover, E protein also suppresses ER stress induced by RSV and drugs (thapsigargin and tunicamycin; DeDiego et al., 2011). Whether the UPR modulating activity is related to the viroporin property of E protein remains to be investigated, but this study explains, at least in part, why SARS-CoV lacking the E protein is attenuated in animal models (Liao et al., 2004; DeDiego et al., 2007).

### IRE1-DEPENDENT DECAY DURING VIRUS INFECTION

Notably, one recent study has demonstrated an alternative function of IRE1. It was found that at the late stage of ER stress, IRE1 mediates non-specific cleavage of membrane-associated mRNA species. This was dubbed IRE1-dependent decay (RIDD) and was proposed to resolve ER stress by reducing the amount of transcripts influx (Hollien et al., 2009). It is intriguing to think of RIDD as a host anti-viral mechanism. During prolonged ER stress induced by infection, non-specific RNase activity of IRE1 may decay the membrane associated viral mRNA. In fact, it has been recently suggested that RIDD is activated during Japanese encephalitis virus (JEV) infection in Neuro2a cells (Bhattacharyya et al., 2014). Interestingly, RIDD specifically degraded known target mRNA transcripts but not JEV RNAs. Also, treatment with IRE1 RNase activity inhibitor suppressed viral replication, indicating that JEV benefits from RIDD activation (Bhattacharyya et al., 2014).

### IRE1 PATHWAY AND INNATE IMMUNITY

Similarly to the integrated stress response, the IRE1 pathway has also been implicated in the innate immune response (Cláudio et al., 2013). Martinon et al. (2010) have shown that in murine macrophages, the IRE1-XBP1 pathway is specifically activated by TLR4 and TLR2. Interestingly, the ER stress and TLR activation synergistically activate IRE1 and induce the production of pro-inflammatory cytokines such as IL-1β and IL-6 (Martinon et al., 2010). Consistently, Hu et al. (2011) have demonstrated that the IRE1-XBP1 pathway is also involved in IFN-β and pro-inflammatory cytokines production in murine DCs induced by polyI:C. Significantly, it has been shown that overexpression of the spliced form of XBP1 enhanced IFN-β production in DCs and significantly suppressed vesicular stomatitis virus infection (Hu et al., 2011). Preliminary results from this group have also found that the activation of IRE1-XBP1 pathway is required for IL-8 induction in cells infected with IBV (unpublished data). On the other hand, the kinase but not the RNAse activity of IRE1 has been associated with ER-stress-induced NF-kB activation (Tam et al., 2012). Under ER stress, IRE1 has been shown to phosphorylate TRAF2, which activates the IκB kinase (IKK) and contributes to its basal activity (**Figure 4**). IKK in turn phosphorylates IκBα and promotes its proteasomal degradation, releasing NF-κB to activate downstream genes (Tam et al., 2012). Taken together, these findings suggest that IRE1 may act synergistically with players in innate immunity and serve as a supplementary sensor and/or signaling factors during coronavirus infection.

## THE ATF6 BRANCH OF UPR

The ER stress sensor ATF6 has an N-terminal cytoplasmic domain, a single transmembrane segment and an ER luminal domain that sense the presence of unfolded/misfolded proteins. Under ER stress, ATF6 is translocated from the ER to the Golgi apparatus and cleaved by protease S1P and S2P (Haze et al., 1999). The cleavage releases the cytosolic basic leucine zipper (bZIP) domain, which translocates into the nucleus and activates genes harboring the ERSE or ERSE II (Yoshida et al., 2001b). The identified target genes of ATF6 include ER chaperones (such as GRP78, GRP94), PDI, and the UPR transcription factors GADD153 and XBP1 (Szegezdi et al., 2006). Previously, it was proposed the ATF6 pathway is mainly pro-survival, as it enhances the ER protein folding capacity to counteract ER stress (Szegezdi et al., 2006). However, recent studies have demonstrated that, under certain circumstances, ATF6-mediated signals may also contribute to ER-stress-induced apoptosis, possibly via activation of CHOP and/or suppression of myeloid cell leukemia sequence 1 (Mcl-1; Gotoh et al., 2002; Nakanishi et al., 2005; Morishima et al., 2011).

The infection of cells by several viruses has been shown to activate the ATF6 pathway, including the Tick-borne encephalitic virus, African swine fever virus (ASFV), West Nile virus (WNV), and HCV (Ambrose and Mackenzie, 2011; Merquiol et al., 2011; Galindo et al., 2012; Yu et al., 2013). In the case of ASFV, ATF6 activation has been shown to modulate ASFV-induced apoptosis and facilitate viral replication (Galindo et al., 2012). For WNV, it has been shown that ATF6 activation promotes efficient WNV replication by suppressing signal transducer and activator of transcription 1 (STAT1) phosphorylation and late-phase IFN signaling (Ambrose and Mackenzie, 2013). The NS4B protein of HCV has been shown to activate ATF6 signaling in cultured cells (Li et al., 2009). Induction of chronic ER stress and adaptation of infected hepatocyte to UPR have been considered important for HCV persistent infection and pathogenesis *in vivo* (Asselah et al., 2010; Merquiol et al., 2011).

Compared with the PERK and IRE1 pathway, the induction of ATF6 pathway during coronaviruses infection has not been deeply investigated. In MHV-infected cells, significant cleavage of ATF6 could be detected starting from 7 h post-infection (Bechill et al., 2008). However, the levels of both full length and cleaved ATF6 protein diminished at later time points during infection. Moreover, activation of ATF6 target genes was not observed at the mRNA level, as determined by luciferase reporter constructs under the control of ERSE promoters (Bechill et al., 2008). It is also unlikely that MHV infection suppresses downstream signaling of the ATF6 pathway, because the reporter induction by overexpressed ATF6 was not inhibited by MHV infection. The authors thus conclude that global translation shutdown via eIF2α phosphorylation prevents accumulation of ATF6 and activation of ATF6 target genes (Bechill et al., 2008). The involvement of ATF6 pathway during infection of other coronaviruses has not been well characterized.

Although the spike proteins of coronaviruses have been considered as the major contributor in ER stress induction, overexpression of SARS-CoV spike protein fails to activate ATF6 reporter constructs (Chan et al., 2006). On the other hand, the accessory protein 8ab of SARS-CoV has been identified to induce ATF6 activation (Sung et al., 2009). The 8ab protein was found in SARS-CoV isolated from animals and early human isolates. In SARS-CoV isolated from humans during the peak of the epidemic, there is a 29-nt deletion in the middle of ORF8, resulting in the splitting of ORF8 into two smaller ORFs, namely ORF8a and ORF8b, which encode two truncated polypeptides 8a and 8b (Guan et al., 2003). ATF6 cleavage and nuclear translocation was observed in cells transfected with SARS-CoV 8ab (Sung et al., 2009). Physical interaction between 8ab and the luminal domain of ATF6 was also demonstrated by co-immunoprecipitation. However, similar experiments have not been performed for the 8a and 8b proteins. Also, further studies using recombinant SARS-CoV lacking 8a, 8b, or 8ab would be required.

## CONCLUSION

Coronaviruses constitute human and animal pathogens that are medically and economically important. Much remains unknown regarding the host–virus interactions during infection. Recent studies have demonstrated that coronaviruse infection induces ER stress in infected cells and activates the UPR. Activation of the PERK pathway (possibly in synergy with PKR and/or other integrated stress response kinases) leads to phosphorylation of eIF2α and a global translation shutdown. At late stage of infection, up-regulation of transcription factor GADD153 likely contributes to coronaviruses induced apoptosis. Activation of the IRE1 pathway induces XBP1 mRNA splicing and expression of downstream UPR genes. Interestingly, IRE1 but not XBP1 is also shown to modulate the JNK and Akt kinase activities, thus protecting infected cells from virus induced apoptosis. The ATF6 pathway is also activated in coronavirus-infected cells, resulting in the up-regulation of chaperon proteins to counteract ER stress.

However, many questions remain to be addressed. First, although the coronaviruses spike proteins are demonstrated to induce ER stress and UPR, detailed mechanisms regarding molecular interactions between the spike proteins and PERK/IRE1/ATF6 have not been determined. Second, it should be noted that the phenotypes observed in cells overexpressing viral proteins may not essentially reflect their physiological functions in the setting of a real infection. Further experiments using recombinant viruses with deletion of or modification in the target viral proteins should be performed to validate these findings (DeDiego et al., 2011). Last but not the least, the three branches of UPR should not be considered functionally independent, but rather as an integrated regulatory network (Ron and Walter, 2007). For example, besides being spliced by IRE1, XBP1 is also transcriptionally activated by PERK and ATF6 (Yoshida et al., 2001a; Calfon et al., 2002). Also, it is difficult to separate the translation shutdown effect mediated by PERK and the induction of UPR genes by PERK and the other two ER stress sensors, as in the studies with MHV (Bechill et al., 2008).

Nonetheless, there are scientific and clinical significance for studies on ER stress and UPR induction during infection with coronaviruses and other viruses. As an evolutionarily conserved and well-characterized stress response pathway, it serves as a perfect model to study host–virus interactions and pathogenesis. Moreover, besides apoptosis, UPR has been recently demonstrated to crosstalk with other major cellular signaling pathways, including MAP kinases pathways, autophagy, and innate immune responses (Yoneda et al., 2001; Ogata et al., 2006; Martinon et al., 2010; Hu et al., 2011; Clavarino et al., 2012). Thus, further investigations on coronavirus-induced UPR may also help identifying new targets for antiviral agents and developing more effective vaccines against coronaviruses.

## Conflict of Interest Statement

The authors declare that the research was conducted in the absence of any commercial or financial relationships that could be construed as a potential conflict of interest.
